# Expression and clinical significance of serum lncRNA H19 in patients with metabolic dysfunction-associated fatty liver disease

**DOI:** 10.1097/MD.0000000000041838

**Published:** 2025-03-14

**Authors:** Ke-Gong Xiong, Jin-Feng Kong, Tai-Shun Lin, Qing-Biao Lin, Li-Fang Chen, Kun-Yu Ke

**Affiliations:** a Department of Hepatology, Mengchao Hepatobiliary Hospital of Fujian Medical University, Fuzhou, China.

**Keywords:** diagnosis, long non-coding RNA H19, metabolic dysfunction-associated fatty liver disease, metabolic risk factors

## Abstract

Long non-coding RNA H19 (lncRNA H19) plays an important role in lipid metabolism, however, its relationship with metabolic dysfunction-associated fatty liver disease (MAFLD) remains unclear. The aim of this study is to investigate the expression and clinical significance of serum lncRNA H19 in patients with MAFLD. This study enrolled patients with MAFLD and a control group of healthy subjects from January 2023 to March 2024. The serum levels of lncRNA H19 were quantified using real-time quantitative polymerase chain reaction. The serum levels of lncRNA H19 in patients with MAFLD were significantly higher compared to the control group (*P* < .05). Moreover, there was a positive correlation between serum lncRNA H19 and body mass index, triglyceride, total cholesterol (TC), low-density lipoprotein cholesterol, fasting blood glucose and uric acid (all *P* < .05). Conversely, a negative correlation was observed between serum lncRNA H19 and high-density lipoprotein cholesterol (HDL-C; *P* = .009). Additionally, significant positive associations were found between serum lncRNA H19 and alanine aminotransferase, aspartate aminotransferase, gamma-glutamyl transpeptidase and liver stiffness measurement(all *P* < .05). The optimal cutoff value of serum lncRNA H19 for diagnosing MAFLD was 1.15, with an area under the curve of the receiver operating characteristic curve of 0.83, and the sensitivity and specificity were observed to be 87.7% and 72.5%, respectively. The lncRNA H19 exhibits associations with metabolic risk factors, liver function, and liver fibrosis, and can serve as a potential diagnostic biomarker for MAFLD.

## 1. Introduction

The term metabolic dysfunction-associated fatty liver disease (MAFLD) was proposed in 2020, replacing the previous designation of nonalcoholic fatty liver disease (NAFLD). It is recognized as a chronic progressive hepatic disorder stemming from metabolic disorders.^[1]^ Due to the escalating prevalence of overweight/obesity and type 2 diabetes mellitus (T2DM), MAFLD has emerged as the predominant chronic liver disease worldwide, exhibiting an incidence rate of up to 25%.^[2]^ Moreover, it can progress to cirrhosis and hepatocellular carcinoma, posing a significant threat to human health. The introduction of the MAFLD designation has enhanced comprehension and facilitated patient classification and management; however, there remains a dearth of specific serological diagnostic markers.

With the rapid advancement of deep sequencing technology, the biological functions of long non-coding RNA (lncRNA) as crucial regulators of gene expression and cell signaling pathways have gained increasing recognition. LncRNA H19 stands out as the pioneering example of an imprinted gene, being the first to be identified and characterized during the pre-genomic era. LncRNA H19 plays a pivotal role in the regulation of numerous biological functions and is implicated in the pathogenesis and progression of diverse liver diseases through distinct mechanisms.^[3]^ The expression of lncRNA H19 was upregulated by fatty acids in hepatocytes and diet-induced fatty liver, leading to the promotion of steatosis and enhancement of lipid accumulation.^[4]^ Moreover, hepatic overexpression of lncRNA H19 facilitated the progression of obstructive cholestatic liver fibrosis.^[5]^ Currently, the expression and clinical significance of lncRNA H19 in patients with MAFLD remain unclear. Therefore, this study aimed to investigate the correlation between serum lncRNA H19 levels in MAFLD patients and metabolic risk factors as well as liver function indicators, while also evaluating its diagnostic value for MAFLD.

## 2. Methods

### 2.1. Study population

A total of 81 patients diagnosed with MAFLD and treated at Mengchao Hepatobiliary Hospital of Fujian Medical University from January 2023 to March 2024 were enrolled. Additionally, 40 subjects who were clinically evaluated and determined not to have MAFLD at the physical examination center from the same hospital during the corresponding period were selected as the control group.

The diagnostic criteria for MAFLD met the 2020 clinical practice guidelines of the Asian Pacific Association for the Study of the Liver.^[6]^ These criteria require evidence of hepatic steatosis along with at least one of the following: body mass index (BMI) ≥ 23 kg/m^2^, T2DM, or metabolic dysfunction (MD) characterized by increased waist circumference, hypertension, hyperlipidemia, hyperglycemia, and insulin resistance.^[6]^ The diagnostic criteria for T2DM were based on the Chinese expert consensus on the management of hypertension in adults with T2DM.^[7]^ Hypertension was defined as systolic blood pressure ≥ 140 mm Hg and/or diastolic blood pressure ≥ 90 mm Hg.^[8]^ Patients with chronic hepatitis B or hepatitis C virus infection, drug-induced liver injury, having autoimmune disorders, parasitic infestations, malignant neoplasms, and severe cardiovascular, neurological, or renal diseases were excluded. All participants provided written informed consent and obtained approval from the Medical Ethics Committee of Mengchao Hepatobiliary Hospital of Fujian Medical University (No. 2023-095-01).

### 2.2. Sample and data collection

A fasting venous blood sample of 5 mL was collected from all subjects and placed in an EDTA anticoagulant tube. The sample was then centrifuged at 3000 rpm for 10 minutes, and the resulting serum was stored at −80°C until RNA extraction. Age, sex, BMI, T2DM, hypertension, albumin (ALB), total bilirubin (TBIL), alanine aminotransferase (ALT), aspartate aminotransferase (AST), gamma-glutamyl transferase (GGT), total cholesterol (TC), high-density lipoprotein cholesterol (HDL-C), low-density lipoprotein cholesterol (LDL-C), triglyceride (TG), fasting plasma glucose (FPG), uric acid (UA), hemoglobin A1C, hypersensitive C-reactive protein levels and liver stiffness measurement (LSM) were obtained from the electronic medical records system used in the hospital. LSM was assessed using FibroScan technology.

### 2.3. Detection of serum lncRNA H19

The expression level of serum lncRNA H19 was quantified using real-time quantitative polymerase chain reaction. Total RNA was extracted from 200 μL serum samples using RNA isolation kit (Takara, China), and cDNA synthesis was performed using a high-capacity cDNA reverse transcription kit (Takara, China) according to the manufacturer’s instructions. Subsequently, qPCR analysis was conducted on the synthesized cDNA utilizing real-time quantitative polymerase chain reaction instrument. The qPCR reaction conditions consisted of an initial activation step at 95°C for 10 minutes, followed by 40 cycles comprising denaturation at 95°C for 15 seconds, annealing at 55°C for 30 seconds, and extension at 72°C for 30 seconds. Primers targeting the sequences of lncRNA H19 and GAPDH genes were procured from Shanghai BioEngineering Company. The relative expression levels of serum lncRNA H19 in MAFLD patients and healthy controls were determined through the calculation of fold change values using the 2^−ΔΔCT^ method.

### 2.4. Statistical analysis

Statistical analysis was performed using SPSS 23 (SPSS Inc., Chicago). For continuous variables, mean ± standard deviation ($\bar{x}\pm s$</mathgraphic>) was used based on the data distribution. Inter-group comparisons were conducted using independent sample *t* test, while multi-group comparisons were analyzed through 1-way analysis of variance. The statistical data were presented as percentages (%) and group comparisons were assessed using χ^2^ test. Pearson correlation was employed to analyze the relationship between serum lncRNA H19 and clinical indicators, and receiver operating characteristic curve analysis was conducted to evaluate the potential diagnostic value of serum lncRNA H19 for MAFLD. Statistical significance was defined as *P* < .05.

## 3. Results

### 3.1. Clinical characteristics of this study cohort

The proportions of BMI ≥ 23 (kg/m^2^) and MD in the MAFLD group were significantly higher compared to the control group (all *P* < .05). Additionally, BMI, ALT, AST, GGT, TG, TC, LDL-C, UA and LSM levels were significantly elevated in the MAFLD group compared to the control group (all *P* < .05), while HDL-C levels were lower than those observed in the control group. Although there was a higher proportion of patients with T2DM in the MAFLD group compared to the control group, this difference did not reach statistical significance (*P* = .183). The 2 groups did not exhibit any significant differences in terms of age, sex, ALB, TBIL, and other variables (all *P* > .05; Table 1).

**Table 1:** Clinical characteristics of this study cohort.

Variables	MAFLD (n = 81)	Control group (n = 40)	*P* value
Age (yr)	42.79 ± 12.96	41.68 ± 12.68	.448
Male	53 (65.4%)	21 (52.5%)	.170
Hypertension	17 (21.0%)	4 (10.0%)	.201
T2DM	15 (18.5%)	3 (7.5%)	.183
MD	24 (29.6%)	4 (10%)	.029
BMI ≥ 23 (kg/m^2^)	69 (85.2%)	26 (65.0%)	.011
BMI (kg/m^2^)	24.93 ± 2.16	22.54 ± 4.17	.001
ALB (g/L)	47.80 ± 3.23	47.18 ± 2.56	.312
TBIL (µmol/L)	13.52 ± 3.65	13.17 ± 4.11	.678
ALT (IU/L)	41.04 ± 15.52	24.48 ± 5.82	<.001
AST (IU/L)	32.11 ± 12.98	20.30 ± 6.46	<.001
GGT (IU/L)	37.62 ± 29.95	20.63 ± 9.90	<.001
TG (mmol/L)	1.60 ± 0.44	1.42 ± 0.25	.006
TC (mmol/L)	5.25 ± 0.79	4.61 ± 0.80	<.001
HDL-C (mmol/L)	1.26 ± 0.32	1.52 ± 0.30	<.001
LDL-C (mmol/L)	3.33 ± 0.77	2.89 ± 0.83	.005
UA (µmol/L)	377.56 ± 90.73	310.95 ± 80.57	<.001
FPG (mmol/L)	5.24 ± 0.80	5.06 ± 0.58	.180
Prothrombin time (s)	11.91 ± 0.99	11.80 ± 0.93	.476
Platelets (10^9^/L)	229.68 ± 39.00	225.93 ± 38.65	.853
LSM (KPa)	6.20 ± 1.75	5.39 ± 1.15	.002

ALB = albumin, ALT = alanine aminotransferase, AST = aspartate aminotransferase, BMI = body mass index, FPG = fasting blood glucose, GGT = gamma-glutamyl transferase, HDL-C = high density lipoprotein cholesterol, LDL-C = low density lipoprotein cholesterol, MAFLD = metabolic dysfunction-associated fatty liver disease, MD = metabolic dysfunction, T2DM = type 2 diabetes mellitus, TBIL = total bilirubin, TC = total cholesterol, TG = triglyceride, UA = uric acid.

### 3.2. Serum lncRNA H19 expression levels

**Figure 1.**
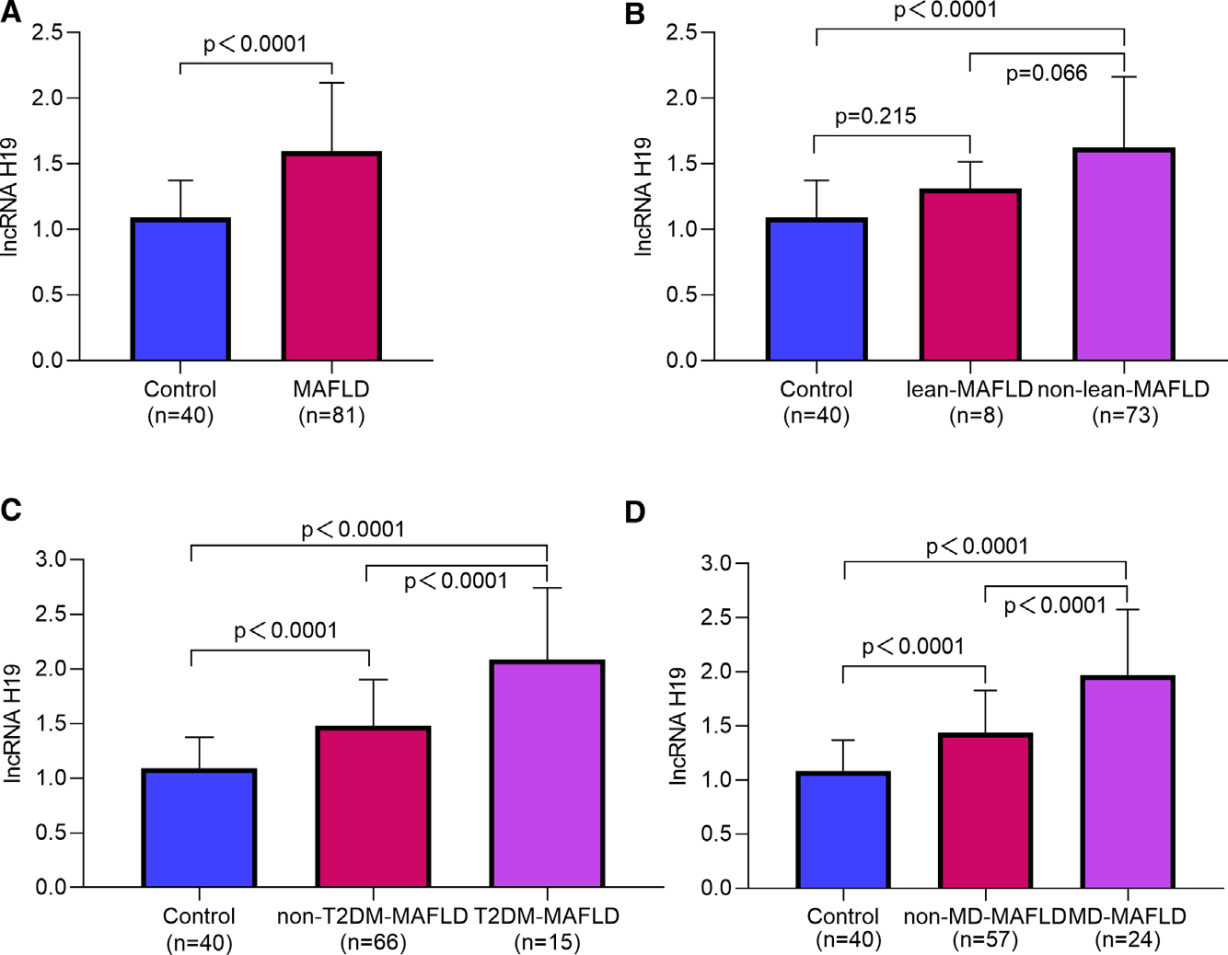
Serum lncRNA H19 expression levels. LncRNA = long non-coding RNA, MAFLD = metabolic dysfunction-associated fatty liver disease, MD = metabolic dysfunction, T2DM = type 2 diabetes mellitus.

The serum level of lncRNA H19 in patients with MAFLD was significantly higher compared to the control group (1.60 ± 0.52 vs 1.09 ± 0.28, *P* < .0001; Fig. 1A). To further determine the levels of serum lncRNA H19 in each subtype of MAFLD, the patients with MAFLD were categorized into 2 groups based on BMI: lean-MAFLD group (BMI < 23 kg/m^2^) consisting of 8 cases (9.9%), and non-lean-MAFLD group (BMI ≥ 23 kg/m^2^) comprising 73 cases (90.1%). The level of serum lncRNA H19 in the non-lean-MAFLD group was significantly higher compared to that in the control group (*P* < .05; Fig. 1B). According to the presence or absence of T2DM, patients with MAFLD were categorized into 2 groups: T2DM-MAFLD group (15 cases, 18.5%) and non-T2DM-MAFLD group (66 cases, 81.5%). Serum lncRNA H19 exhibited a gradual increase in all 3 groups (all *P* < .05; Fig. 1C). Based on the presence or absence of MD, MAFLD patients were categorized into MD-MAFLD group (24 cases, 29.6%) and non-MD-MAFLD group (57 cases, 70.4%). Furthermore, serum levels of lncRNA H19 also demonstrated a progressive elevation across the 3 MD groups (all *P* < .05; Fig. 1D).

### 3.3. Correlation of lncRNA H19 with metabolic risk factors

The Pearson correlation analysis revealed a positive association between serum lncRNA H19 levels and metabolic risk factors (BMI, TG, TC, LDL-C, FPG, and UA) in MAFLD patients (all *P* < .05), while a negative correlation was observed with HDL-C levels (*P* = .009; Table 2).

**Table 2:** Correlation of lncRNA H19 with metabolic risk factors.

Variables		1	2	3	4	5	6	7
1 lncRNA H19	Correlation							
	*P*							
2 BMI	Correlation	0.252**						
	*P*	.005						
3 TG	Correlation	0.297**	0.101					
	*P*	.001	.269					
4 TC	Correlation	0.344**	0.356**	0.084				
	*P*	0	0	.359				
5 HLD-C	Correlation	−0.382**	−0.212*	0.103	−0.273**			
	*P*	0	.019	.259	.002			
6 LDL-C	Correlation	0.323**	0.165	0.155	0.386**	−0.087		
	*P*	0	.070	.090	.000	.345		
7 UA	Correlation	0.364**	0.193*	0.149	0.299**	−0.242**	0.227*	
	*P*	0	.034	.102	.001	.008	.012	
8 FBG	Correlation	0.208*	0.002	0.198*	0.094	0.045	0.077	0.141
	*P*	.022	.982	.029	.306	.623	.402	.123

BMI = body mass index, FPG = fasting blood glucose, HDL-C = high density lipoprotein cholesterol, LDL-C = low density lipoprotein cholesterol, lncRNA = long non-coding RNA, TC = total cholesterol, TG = triglyceride, UA = uric acid.

* *P* *<* .05.

** *P* < .01.

### 3.4. Correlation of lncRNA H19 with clinical indicators

The Pearson correlation analysis revealed significant positive associations between serum lncRNA H19 levels and liver function indicators (ALT, AST, and GGT) as well as the fibrosis indicator (LSM) in the study population (all *P* < .05; Table 3).

**Table 3:** Correlation of lncRNA H19 with clinical indicators.

Variables		1	2	3	4	5	6
1 LncRNA H19	Correlation						
	*P*						
2 ALB	Correlation	−0.144					
	*P*	.115					
3 TBIL	Correlation	0.039	−0.135				
	*P*	.674	.140				
4 ALT	Correlation	0.497**	0.052	0.072			
	*P*	0	.570	.429			
5 AST	Correlation	0.484**	0.002	−0.036	0.808**		
	*P*	.000	.985	.692	0		
6 GGT	Correlation	0.302**	0.061	−0.021	0.410**	0.467**	
	*P*	.001	.509	.819	0	0	
7 LSM	Correlation	0.509**	−0.238**	0.134	0.283**	0.189*	0.224*
	*P*	0	.008	.142	.002	.038	.013

ALB = albumin, ALT = alanine aminotransferase, AST = aspartate aminotransferase, GGT = gamma-glutamyl transferase, lncRNA = long non-coding RNA, LSM = liver stiffness measurement, TBIL = total bilirubin.

* *P* < .05.

** *P* < .01.

### 3.5. Diagnostic value of lncRNA H19 in MAFLD

**Figure 2.**
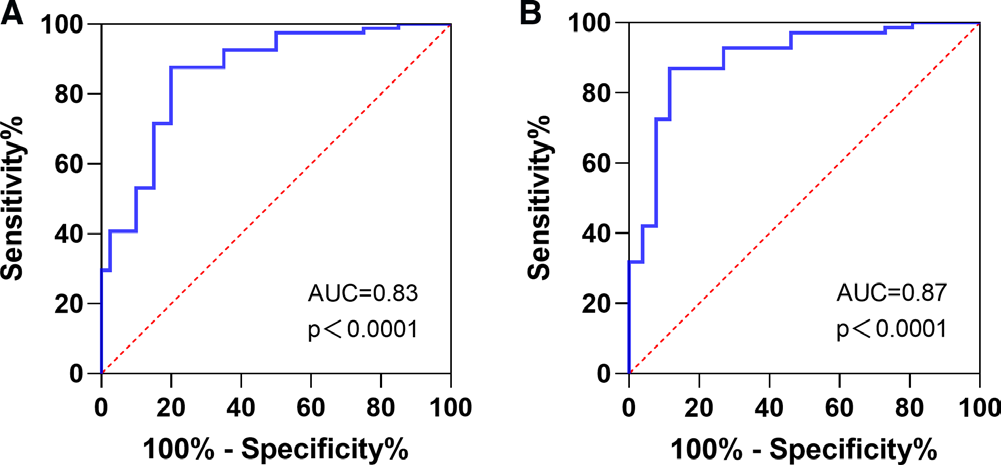
Diagnostic value analysis of lncRNA H19 in MAFLD. lncRNA = long non-coding RNA, MAFLD = metabolic dysfunction-associated fatty liver disease.

The receiver operating characteristic curve analysis revealed that the optimal cutoff value of serum lncRNA H19 for diagnosing MAFLD was determined to be 1.15, with an area under the curve (AUC) of 0.83. The sensitivity and specificity were found to be 87.7% and 72.5%, respectively (Table 4 and Fig. 2A). Further subgroup analysis among individuals with BMI ≥ 23 kg/m^2^ demonstrated that the optimal cutoff value of serum lncRNA H19 for diagnosing MAFLD remained at 1.25, while the AUC improved to 0.87, with a sensitivity of 87.0% and specificity of 76.9% (Table 4 and Fig. 2B).

**Table 4:** Diagnostic value of lncRNA H19 in MAFLD.

Variables	Cutoff value	Sen (%)	Spe (%)	PPV (%)	NPV (%)	Youden index (%)
lncRNA H19*	1.15	87.7	72.5	86.6	74.4	60.2
lncRNA H19**	1.25	87.0	76.9	90.9	69.0	63.9

lncRNA = long non-coding RNA, MAFLD = metabolic dysfunction-associated fatty liver disease, NPV = negative predictive value, PPV = positive predictive value, Sen = sensitivity, Spe = specificity, Youden index = sensitivity + specificity − 1.

* All subjects.

** Subjects with BMI ≥23 kg/m^2^.

## 4. Discussion

The pathogenesis of MAFLD remains elusive, and there is a dearth of serum-specific diagnostic markers. In this study, we conducted the first investigation into the expression of lncRNA H19 in MAFLD patients to explore its clinical significance. Our findings revealed a significant association between elevated serum levels of lncRNA H19 in MAFLD patients and metabolic risk factors as well as liver-related indicators, suggesting its potential utility as a diagnostic marker for MAFLD.

The pathogenesis of MAFLD is intricate, with emerging evidence highlighting the pivotal role of metabolic disorders in its etiology.^[9]^ The lncRNA H19 has garnered increasing attention owing to its wide-ranging physiological and pathological functionalities. While the basal expression level of lncRNA H19 in the liver remains low under normal physiological conditions, it exhibits a propensity for up-regulation during pathological states.^[10]^ Previous animal studies^[11,12]^ have demonstrated that lncRNA H19 expression is upregulated during the development of oleic acid-induced steatosis and high-fat diet-induced NAFLD mouse models, leading to lipid accumulation in hepatocytes. Conversely, the downregulation of lncRNA H19 expression results in reduced hepatic lipid accumulation. Furthermore, additional investigations have demonstrated that lncRNA H19 exerts a hepatoprotective effect by directly modulating the miR-130a/PPARγ axis, thereby attenuating hepatic lipid accumulation and potentially contributing to the pathogenesis of NAFLD.^[13]^ The expression of lncRNA H19 in the liver of db/db mice was downregulated, leading to an increased binding of p53 on the FoxO1 promoter and subsequently promoting FoxO1 transcription. FoxO1 was an important transcriptional regulator of gluconeogenesis. The knockout of lncRNA H19 in the liver of normal mice resulted in elevated blood glucose levels, impaired glucose tolerance, and upregulated expression of gluconeogenic genes.^[14]^ Relevant investigation has also demonstrated the involvement of lncRNA H19 in the regulation of gluconeogenesis.^[15]^ These findings suggest that lncRNA H19 plays a crucial role in lipid and glucose metabolism. From a clinical perspective, this study revealed that serum levels of lncRNA H19 were elevated in patients with MAFLD compared to the control group. Furthermore, these levels exhibited significant correlations with various metabolic risk factors, including BMI, TG, TC, HDL-C, LDL-C, FPG, and UA. It is suggested that lncRNA H19 is closely associated with the development of MAFLD.

Despite the recognized significance of lncRNA H19 in diet-induced hepatic steatosis and glucose metabolism, it remains unclear whether and how lncRNA H19 is involved in MAFLD disease progression. In this study, a significant correlation was observed between lncRNA H19 and liver function markers, including ALT, AST, and GGT. Additionally, an association between lncRNA H19 and LSM, an indicator of liver fibrosis, was identified. The pathogenesis of liver fibrosis is closely associated with liver inflammation. Prolonged and dysregulated chronic inflammation facilitates this process by inducing the production of anti-inflammatory and pro-fibrotic cytokines and chemokines. Irrespective of etiology, hepatic overexpression of lncRNA H19 significantly facilitates liver fibrosis, whereas ablation of lncRNA H19 confers protection against this pathological process.^[16,17]^ Hepatic fibrosis is a reparative process characterized by excessive deposition of extracellular matrix (ECM) due to the activation of hepatic stellate cells (HSCs). A recent study has demonstrated that elevated expression of lncRNA H19 in various chronic liver diseases contributes to liver fibrosis by directly interacting with enhancer of zeste homolog 2, thereby facilitating the activation of HSCs.^[18]^ The findings of this study suggest that lncRNA H19 is not only implicated in the pathogenesis of MAFLD but also plays a significant role in its progression.

MAFLD can result in liver fibrosis, cirrhosis, and hepatocellular carcinoma, as well as increase the risk of extrahepatic diseases such as cardiovascular disease. With the rising global incidence of MAFLD, it poses an escalating health burden. However, MAFLD lacks specific clinical symptoms and currently lacks a standardized universal screening method. Although various noninvasive tests are employed to assess MAFLD, each has its limitations.^[19]^ Consequently, an increasing amount of research is being conducted to investigate user-friendly, pragmatic, and dependable approaches for predicting MAFLD, a pressing concern that necessitates attention in clinical practice. Previous studies have demonstrated the involvement of serological markers, including adipocyte-secreted lipoprotein lipase, leptin, and macrophage scavenger receptor 1, in the pathogenesis of MAFLD through their regulation of hepatic lipid accumulation, insulin resistance, and fibrosis.^[20–22]^ Another study has also demonstrated that clinical indicators, including BMI, waist circumference, visceral fat index, lipid accumulation products, and triglyceride glucose index, play a crucial role in identifying the risk of MAFLD.^[23]^ This study demonstrates that serum lncRNA H19 exhibits excellent diagnostic performance for MAFLD, with an AUC of 0.83, a sensitivity of 87.7%, and a specificity of 72.5%. Furthermore, the subgroup analysis revealed even higher diagnostic accuracy in individuals with BMI ≥ 23 kg/m^2^, with an AUC of 0.87, sensitivity of 87.0%, and specificity of 76.9%. These findings suggest that serum lncRNA H19 holds promise as a serological biomarker for early detection of MAFLD.

The study also has certain limitations. Firstly, it should be noted that this study is conducted at a single center, and therefore the findings necessitate validation through multi-center studies with a larger sample size. Secondly, as this study falls under the category of clinical research, further investigation into the underlying participation mechanism is warranted. Furthermore, in this study, none of the participants underwent liver tissue biopsy; only noninvasive indicators were utilized to assess liver inflammation and fibrosis.

In conclusion, lncRNA H19 is implicated in the pathogenesis and progression of MAFLD, rendering it a promising serum diagnostic marker and potential therapeutic target for this disease. However, further fundamental research is warranted to elucidate the precise mechanism underlying the involvement of lncRNA H19 in MAFLD.

## Author contributions

**Conceptualization:** Ke-Gong Xiong, Kun-Yu Ke.

**Data curation:** Ke-Gong Xiong, Jin-Feng Kong.

**Formal analysis:** Ke-Gong Xiong, Tai-Shun Lin.

**Funding acquisition:** Ke-Gong Xiong.

**Investigation:** Ke-Gong Xiong, Jin-Feng Kong, Tai-Shun Lin, Qing-Biao Lin.

**Project administration:** Jin-Feng Kong, Li-Fang Chen, Kun-Yu Ke.

**Resources:** Tai-Shun Lin, Qing-Biao Lin, Li-Fang Chen.

**Supervision:** Kun-Yu Ke.

**Validation:** Jin-Feng Kong.

**Writing – original draft:** Ke-Gong Xiong.

**Writing – review & editing:** Jin-Feng Kong, Kun-Yu Ke.
